# Profiling of Polar Lipids in Marine Oleaginous Diatom *Fistulifera solaris* JPCC DA0580: Prediction of the Potential Mechanism for Eicosapentaenoic Acid-Incorporation into Triacylglycerol

**DOI:** 10.3390/md12063218

**Published:** 2014-05-28

**Authors:** Yue Liang, Yoshiaki Maeda, Tomoko Yoshino, Mitsufumi Matsumoto, Tsuyoshi Tanaka

**Affiliations:** 1 Division of Biotechnology and Life Science, Institute of Engineering, Tokyo University of Agriculture and Technology, 2-24-16, Naka-cho, Koganei, Tokyo 184-8588, Japan; E-Mails: y_liang@cc.tuat.ac.jp (Y.L.); y_maeda@cc.tuat.ac.jp (Y.M.); y-tomoko@cc.tuat.ac.jp (T.Y.); 2 Biotechnology Laboratory, Electric Power Development Co., Ltd., 1, Yanagisaki-machi, Wakamatsu-ku, Kitakyusyu 808-0111, Japan; E-Mail: mitsufumi_matsumoto@jpower.co.jp; 3 Japan Science and Technology Agency (JST), Core Research for Evolutionary Science and Technology (CREST), 5, Sanbancho, Chiyoda-ku, Tokyo 102-0075, Japan

**Keywords:** marine oleaginous diatom, *Fistuliferasolaris*, lipid synthesis, eicosapentaenoic acid (EPA), PC-based acyl-editing

## Abstract

The marine oleaginous diatom *Fistulifera* *solaris* JPCC DA0580 is a candidate for biodiesel production because of its high lipid productivity. However, the substantial eicosapentaenoic acid (EPA) content in this strain would affect the biodiesel quality. On the other hand, EPA is also known as the essential health supplement for humans. EPAs are mainly incorporated into glycerolipids in the microalgal cell instead of the presence as free fatty acids. Therefore, the understanding of the EPA biosynthesis including the incorporation of the EPA into glycerolipids especially triacylglycerol (TAG) is fundamental for regulating EPA content for different purposes. In this study, in order to identify the biosynthesis pathway for the EPA-containing TAG species, a lipidomic characterization of the EPA-enriched polar lipids was performed by using direct infusion electrospray ionization (ESI)-Q-TRAP-MS and MS/MS analyses. The determination of the fatty acid positional distribution showed that the *sn*-2 position of all the chloroplast lipids and part of phosphatidylcholine (PC) species was occupied by C16 fatty acids. This result suggested the critical role of the chloroplast on the lipid synthesis in *F. solaris*. Furthermore, the exclusive presence of C18 fatty acids in PC highly indicated the biosynthesis of EPA on PC. Finally, the PC-based acyl-editing and head group exchange processes were proposed to be essential for the incorporation of EPA into TAG and chloroplast lipids.

## 1. Introduction

Over the past several decades, biofuel production has attracted much interest due to the approaching exhaustion of fossil fuels and their negative impacts on climate. Microalgae have been recognized as promising biofuel producers due to their advantages of higher biomass productivity and non-competition with foods [1,2,3]. Recent studies have discovered a number of microalgae possessing high content of lipids [4,5,6,7,8,9].

In addition to lipid content, the unsaturation degree of microalgal lipids, which directly determines the fuel quality, is another critical factor for biodiesel application [10]. High content of polyunsaturated fatty acid (PUFA) will result in low oxidative stability of the final product [11]. The PUFA (≥ 4 double bonds) content in biodiesel is limited to less than 1% by the EN 14214 standards in Europe. However, on the other hand, omega 3-long chain PUFAs (ω3-LCPUFAs), e.g., eicosapentaenoic acid (EPA, C20:5n3) and docosahexaenoic acid (DHA, C22:5n3) are essential health supplements for humans [12]. The growing market of ω3-LCPUFAs has made it impossible to maintain a sustainable supply from the conventional source of fish oil. Therefore, the research in polyunsaturated lipid synthesis in microalgae thus receives much attention. Some microalgal strains, which were determined to produce high content of EPA and DHA [13], are the potential alternative source of ω3-LCPUFAs. The understanding of the biosynthesis pathway for ω3-LCPUFA and the assembly of ω3-LCPUFA into lipids is the essential task to control ω3-LCPUFA content in microalgae by means of metabolic engineering.

Among eukaryotic microalgae, a pennate diatom, *Phaeodactylum tricornutum* has been used as one of the model organisms for studies in ω3-LCPUFA synthesis [14,15] due to its high content of EPA [16], available genome information [17] and easy transformation [18,19]. In *P. tricornutum*, metabolic labeling analyses revealed that phosphatidylcholine (PC) was involved in the series of fatty acid-desaturation for EPA synthesis [15]. However, the mechanism how EPA is incorporated into triacylglycerol (TAG, a main source of biodiesel) has not yet thoroughly been discussed in any microalgae. Furthermore, the lipid content in *P. tricornutum* is moderate [5]; thus, this microalga may not be the best lipid producer for biodiesel production. In addition, the knowledge of polyunsatulated lipid synthesis in other diatoms is poorly accumulated.

In this study, we focused on another marine pennate diatom, *Fistulifera* *solaris* (formerly *Fistulifera* sp.) JPCC DA0580, which has been identified in our lab [20] as an oleaginous microalga and can accumulate oil up to nearly 60% of dried cell weight (DCW) [9,21]. *F. solaris* also maintains substantial amount of EPA [9,22]. Owing to the achievements on mass cultivation [9,23] genome sequencing (partially published [24]) and genetic transformation [25], we propose that this strain is one of the ideal candidates for either biofuel or EPA production. Recently, the lipidomic analysis targeting neutral lipids, and the desaturation process for EPA synthesis in *F. solaris* has been reported [22,26], while the exact substrates for desaturation and the process of EPA-incorporation into TAG have not yet been addressed. In this study, the fatty acid composition and positional distribution of major polar lipid species were determined. The distribution of EPA and its precursor fatty acids in polar lipids was analyzed with direct infusion electrospray ionization (ESI)-Q-TRAP-MS/MS in order to deeply understand the polyunsatulated lipid synthesis in *F. solaris*. The results suggest that EPA was desaturated on PC and the PC-based acyl-editing and head group exchange processes may contribute to the EPA-incorporation into TAG.

## 2. Results and Discussion

### 2.1. Polar Lipid Profile of Fistulifera solaris JPCC DA0580

**Figure 1:**
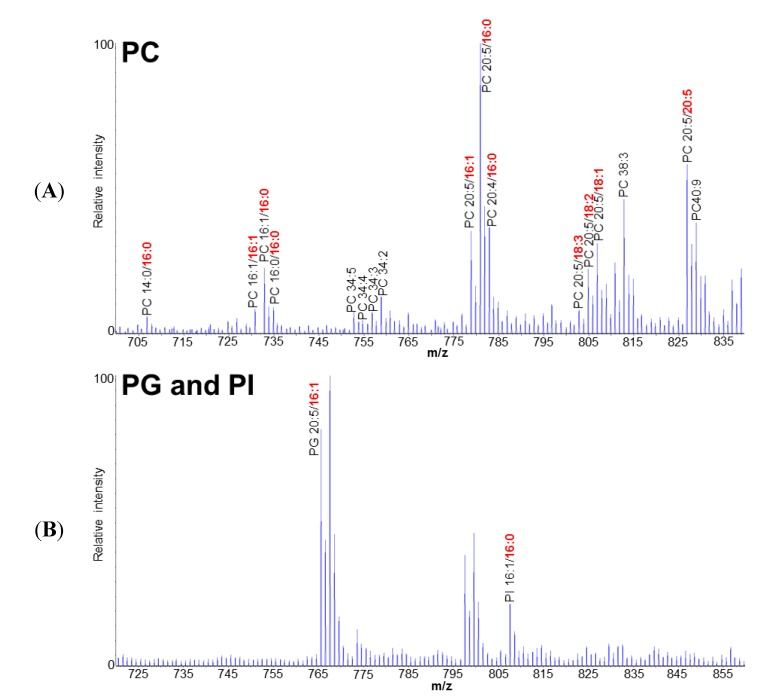
The representative MS spectra of phosphatidylcholine (PC) (**A**); phosphatidylglycerol (PG) (**B**), and phosphatidylinositol (PI) (**B**) profile of *Fistulifera* sp. Fatty acids in the *sn*-2 position are represented in bold and red.

**Figure 2:**
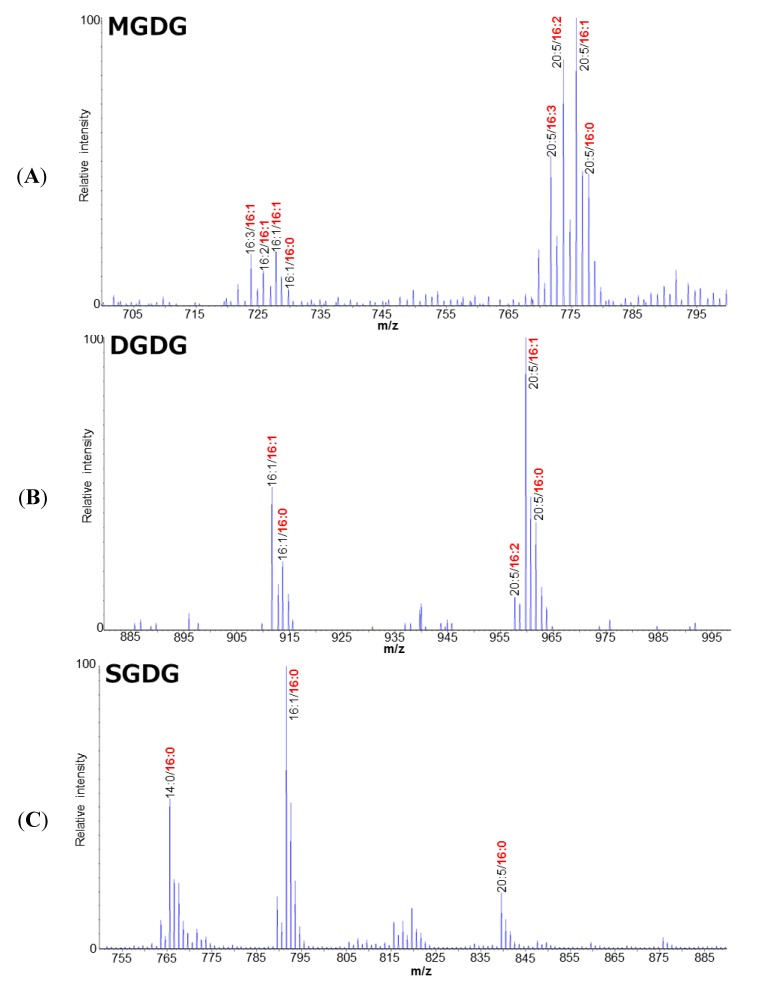
The representative MS spectra of the monogalactosyldiacylglycerol (MGDG) (**A**); digalactosyldiacylglycerol (DGDG) (**B**); and sulfoquinovosyldiacylglycerol (SQDG) (**C**) profile of *Fistulifera* sp. Fatty acids in the *sn*-2 position are represented in bold and red.

In order to examine how EPA is synthesized and incorporated into TAGs in *F. solaris*, the polar lipid profile including the information of fatty acid composition and positional distribution was elucidated by direct infusion ESI-Q-TRAP-MS/MS, through which the intact molecular structures of lipids can be determined. In the phospholipid (PL) fraction, 14 types of lipids including 11 phosphatidylcholines (PCs), 2 phosphatidylglycerol (PGs), and 1 phosphatidylinositol (PI) were identified (Figure 1). Phosphatidylethanolamine (PE) was not detected in the PL fraction, indicating the absence of PE in *F. solaris*. In the glycolipid (GL) fraction, 16 types of lipids including 8 monogalactosyldiacylglycerol (MGDGs), 5 digalactosyldiacylglycerol (DGDGs), and 3 sulfoquinovosyldiacylglycerol SQDGs were identified (Figure 2).

**Figure 3:**
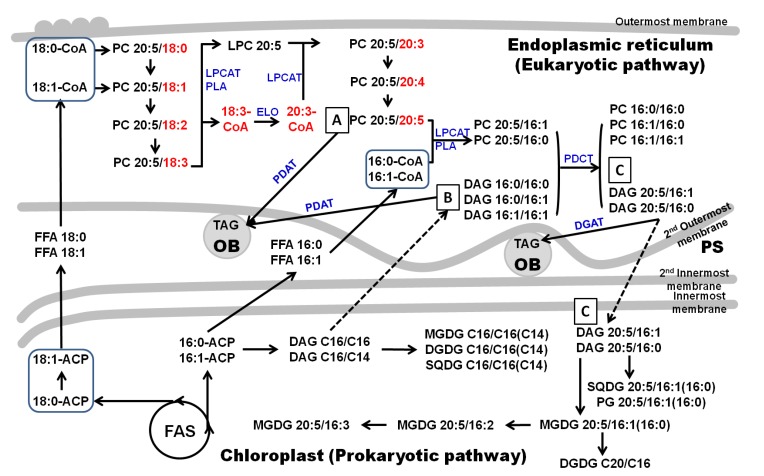
The scheme of the putative polar lipids and eicosapentaenoic acid (EPA) synthesis pathway in *Fistulifera* *solaris*. The plastid in this strain is considered to be surrounded by four membranes. Fatty acids and the prokaryotic lipids are *de novo* synthesized in the primary plastid. The eukaryotic lipids are *de novo* synthesized in the endoplasmic reticulum. The EPA was synthesized through the sequential desaturation of C18 fatty acid on PC. The acyl-editing and head group exchange processes on PC could not only provide EPA-CoA and EPA containing DAGs for the synthesis of other EPA-containing lipids but also promote the synthesis of the PC species maintain two C16 fatty acids. TAG in this figure is specified as the EPA-containing prokaryotic TAG. Arrow-headed lines represent synthesis, while dashed arrow-headed line represents transportation. OB, oil body; PS, periplastidal space; FAS, fatty acid synthesis; PLA, phospholipase A; LPCAT, acyl-CoA, lyso-phosphatidylcholine acyltransferase; PDCT, phosphatidylcholine diacylglycerol cholinephosphotransferase; FFA, free fatty acid; PDAT, phospholipid, diacylglycerol actyltransferase; DGAT, acyl-CoA, diacylglycerolacyltransferase; ELO,elongase.

There are two distinct pathways for glycerolipid assembly in higher plants [27] and microalgae [28], *i.e.*, a prokaryotic pathway and a eukaryotic pathway. In diatoms, the chloroplast is enveloped with four membranes (outermost, second outermost, second innermost and innermost membranes, see also Figure 3). The innermost and second innermost membranes are actually corresponding to the two membranes of the primary chloroplast where glycerolipids are synthesized through the prokaryotic pathway. The space between the second outermost membrane and the outermost membrane represents the endoplasmic reticulum (ER, also known as chloroplast-ER; CER) where the eukaryotic pathway is responsible for the lipid synthesis. The lipids assembled through the prokaryotic pathway have a C16 fatty acid at the *sn-*2 position, while those through the eukaryotic pathway have a C18 or C20 fatty acid at the *sn-*2 position.

In *F. solaris*, the *sn-*2 position of major MGDGs, DGDGs, SQDGs, PGs and PI species was exclusively occupied by a C16 fatty acid. A part of PC species also possessed a C16 fatty acid at the *sn-*2 position (Figure 1A). In addition, the *sn-*2 position in most TAGs was also occupied by C16 fatty acids [22]. This specific distribution of C16 at the *sn-*2 position in large proportion of lipids was also reported in *P. tricornutum* [29,30]. These results indicate that the prokaryotic pathway in the chloroplast plays an essential role in the storage lipid TAG and chloroplast lipids including MGDG, DGDG, SQDG, and PG in diatoms. PC had both C16 and C18 (C20) fatty acids at the *sn*-2 position (Figure 1A), suggesting that both prokaryotic (in the chloroplast) and eukaryotic (in the ER) pathways could contribute to PC synthesis. This characteristic feature of PC has previously been confirmed only in *P. tricornutum* [30], and has not been found in higher plants and green algae, implying that this duplicated origin of PC could be a unique for diatoms. Furthermore, an *F. solaris*-specific feature was also found. The eukaryotic type of GLs which have been found in *P. tricornutum* [30] (e.g., MGDG 20:5/18:4, MGDG 20:5/20:5, MGDG 20:5/18:2, SQDG 20:5/18:4 and SQDG 22:6/18:1 (*sn*-1/*sn*-2)), were not detectable in this study.

### 2.2. Putative Pathways for the PUFA Synthesis in Fistulifera solaris

Another important finding of our analysis is that C18 fatty acids (e.g., C18:1, C18:2 and C18:3) were found only on PCs (Figure 1A). More specifically, C18 fatty acids are distributed only at the *sn*-2 position of PCs, indicating that these PCs are assembled through the eukaryotic pathway in ER. Since these C18 fatty acids are essential precursors for EPA synthesis [26], we propose that EPA synthesis in *F. solaris* was, at least partially, performed on PC in ER (Figure 3). EPA synthesis in ER is consistent with our previous study where most enzymes involved in the EPA synthesis are localized in the ER [26,31]. Furthermore, the involvement of PC in the EPA synthesis is also consistent with the previous metabolic labeling study on *P. tricornutum* [15], in which it was demonstrated that the C18:1 was desaturated on PC to C18:3 or C18:4, the subsequent elongation of the C18:3 and C18:4 to the C20:3 and C20:4 occurred on fatty acid-CoA, and finally, the C20:3 and C20:4 were desaturated to EPA on PC. These results suggest that diatoms may share the EPA synthesis mechanism in which PCs play a central role, although we have previously demonstrated that the EPA synthesis pathway in *F. solaris* was simpler than in *P. tricormutum* [26].

### 2.3. EPA Incorporation into TAG through the Acyl-Editing Process on PC

The EPA-containing TAGs in *F. solaris* mainly contain a C16 fatty acid at the *sn*-2 position which suggests the prokaryotic origination of those TAGs [22]. These TAGs can be theoretically synthesized by two pathways: the diacylglycerol acyltransferase (DGAT)-mediated *de novo* synthesis (the so called Kennedy pathway) and the phospholipid diacylglycerol acyltransferase (PDAT)-mediated membrane conversion. The DGAT catalyzes the acyltransfer reaction which incorporates the fatty acid at the fatty acid-CoA to the *sn*-3 or *sn*-1 position of the diacylglycerol (DAG) to generate TAG, while the PDAT catalyzes the acyltransfer reaction that incorporates the fatty acid at the *sn*-2 position of PC to the *sn*-3 or *sn*-1 position of DAG to generate TAG. For both enzymes, the substrate DAG is used as the backbone for TAG synthesis. In *F. solaris*, the exclusive existence of C14, C16 and C20:5 in the EPA-containing TAGs [22] strongly indicated that the major precursors for the synthesis of these specific TAGs could be DAG C16/C16 (*sn*-1*/sn*-2) (Figure 3; compound B) or DAG EPA/C16 (*sn*-1*/sn*-2) (Figure 3; compound C) because C14 fatty acid presented in a minor percentage in this strain.

The most simple and reasonable pathway for the synthesis of EPA-containing prokaryotic TAG could be the PDAT-mediated acyltransfer with DAG C16/C16 (Figure 3; compound B) and PC EPA/EPA (Figure 3; compound A). Another possible pathway is DGAT-mediated acyltransfer with DAG EPA/C16 (*sn*-1/*sn*-2) (Figure 3; compound C) and fatty acid-CoA. We believe these two pathways are major contributors to incorporate EPA into TAG.

**Figure 4:**
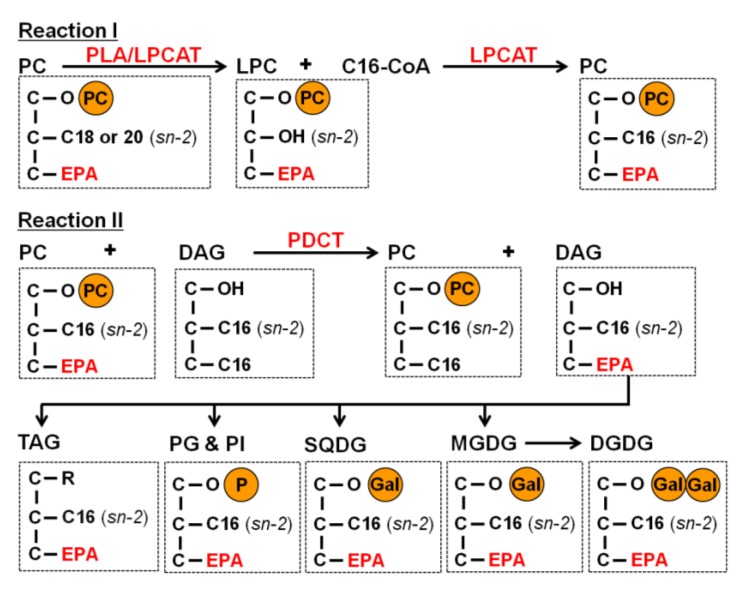
The scheme of the proposed PC-based acyl-editing and head group exchange processes, which are catalyzed by PLA, LPCAT, and PDCT, for the synthesis of prokaryotic EPA-containing TAG, MGDG, DGDG, SQDG, PG and PI in *F. solaris*.

Thus far, the mechanism for the DAG EPA/C16 synthesis has never been discussed in any microalgae, but is important to be elucidated for controlling EPA-incorporation into TAG. One possibility is the *de novo* synthesis of DAG EPA/C16. In this mechanism, the EPA can be released from the PC in the ER to generate EPA-CoA. The generated EPA-CoA could then be transported into the chloroplast to generate EPA-ACP. Finally, the EPA on EPA-ACP is incorporated into DAG EPA/C16 in the chloroplast. However, this circuitous pathway can hardly be considered as reasonable. Therefore, we propose another pathway for the synthesis of DAG EPA/C16; *i.e.*, the PC-based acyl-editing and head group exchange processes, which are recently demonstrated in higher plants to be essential for the incorporation of PUFA into TAG [32,33]. EPA-incorporation through this process is summarized in Figure 3. Three key enzymes are involved in these processese, the phospholipase A (PLA), acyl-CoA: lyso-phosphatidylcholine acyltransferase (LPCAT), and the phosphatidylcholine diacylglycerol cholinephosphotransferase (PDCT). The PLA catalyzes the reaction to degrade the PC into a lyso-PC (LPC) and the free fatty acid which was then converted to acyl-CoA by acyl-CoA synthetases. LPCAT catalyzes the esterification of the *sn*-2 position of a lyso-PC (LPC) to generate PC, as well as the reverse reaction [33]. PDCT catalyzes the transfer of the phosphocholine head group from PC to DAG to generate new species of PC and DAG. The eukaryotic type of PCs which maintain an EPA at the *sn-*1 position and a C18 or C20 fatty acid at the *sn*-2 position, which were abundantly detected (Figure 1A), can be served as the starting materials to produce DAG EPA/C16. The *sn*-2 positions of these EPA-containing eukaryotic PCs are then converted to the most abundant C16 through the PLA and LPCAT mediated acyl-editing process (Figure 4, Reaction I). The generated EPA-containing PCs could subsequently transfer their head group to the predominant DAG C16/C16 through the PDCT mediated head group exchange process to generate the DAG EPA/C16 (*sn*-1/*sn*-2) and PC C16/C16 (Figure 4, Reaction II). The DAG EPA/C16 (*sn*-1/*sn*-2) can be utilized for the generation of EPA-containing TAGs as well as the EPA-containing prokaryotic MGDG, DGDG, SQDG, PG, and PI (Figure 4).

## 3. Experimental Section

### 3.1. Materials

The marine oleaginous diatom, *Fistulifera* sp. JPCC DA0580 was isolated by and identified in our group [21]. Silica gel 60 pre-coated thin-layer chromatography plates were from Merck (Darmstadt, Germany). The Supelclean™ LC-Si SPE Tube (100 mg, 1 mL) was purchased from Sigma Aldrich (Tokyo, Japan). LC-MS grade methanol, LC-MS grade formic acid, and HPLC grade chloroform were purchased from Wako (Tokyo, Japan). All other reagents were of the highest commercial grade available.

### 3.2. Strain and Culture Conditions

The cells were cultured as described previously [22]. In order to enhance the polar lipids recovery in *F. solaris* JPCC DA0580, the nutrition-rich 10f medium which contained 10-fold more nutrition components than f medium (150 mg NaNO_3_, 12 mg Na_2_HPO_4_·2H_2_O, 1 μg vitamin B_12_, 1 μg biotin, 200 μg thiamine HCl, 20 mg Na_2_SiO_3_·9H_2_O, 8.8 mg Na_2_EDTA, 6.32 mg FeCl_3_·6H_2_O, 24 μg CoSO_4_·5H_2_O, 42 μg ZnSO_4_·7H_2_O, 0.36 mg MnCl_2_·4H_2_O, 140 μg CuSO_4_·5H_2_O, and 14 μg Na_2_MoO_4_·2H_2_O per liter of artificial seawater) [34] was selected to eliminate the intensive neutral lipid accumulation [22]. The cells were cultured with the initial cell concentration of 1.0 × 10^6^ cells∙mL^−1^ in flat-shape flasks (1.5 L). The growth curve was shown in Supplementary Figure S1. The temperature was maintained at 25 ± 1 °C, and continuous illumination was applied at 200 μmol·m^−2^·s^−1^. The cultures were bubbled with sterile air containing 2% CO_2_ at a flow rate of 0.8 L/min. After 180 h of cultivation, the cells were harvested by centrifugation at 8500× *g* for 15 min and freeze-dried.

### 3.3. Lipid Extraction

Total lipids were extracted from the lyophilized cells by two-step chloroform/methanol extraction described by Ejsing *et al.* [35] with some modifications. In general, 25 mg of the lyophilized microalgal cells were re-suspended in 5 mL of chloroform/methanol (20:1 v/v) and disrupted by 15 min-sonication. Phase separation was induced by adding distillated water (1 mL). After centrifugation at 800× *g* for 10 min, the lower phase was collected. The remaining aqueous sample material was processed with the extraction procedure again with 5 mL chloroform/methanol (2:1, v/v). The lower organic phase was collected and combined with the formerly collected organic phase. The lipid extracts were dried under argon gas and stored at −30 °C.

### 3.4. Lipid Fractionation

In order to avoid the unwanted overlap of isotopic ion peaks in the direct infusion ESI-Q-TRAP MS analysis, the solid phase extraction (SPE) was carried out to separate the total lipid extract. The Supelclean™ LC-Si SPE Tube (100 mg, 1 mL) was used to fractionate the total lipids into neutral lipids (NLs), glycolipids (GLs), and phospholipids (PLs) according to the method of Popovich *et al.* [36] with minor modifications. The columns were conditioned with 4 mL of hexane. The crude lipid extracts were dissolved in 200 μL of chloroform and loaded into the column. NLs were eluted with 4 mL of chloroform, and GLs were eluted with 4 mL of acetone/methanol (9:1 v/v), finally PLs were eluted with 4 mL of methanol. Each lipid fraction was collected into a glass vial and dried under argon gas. The efficiency of SPE separation was verified by thin-layer chromatography (TLC). Briefly, each lipid fraction was analyzed by TLC. The neutral lipids were developed by *n*-hexane:diethyl ether:acetic acid 70:30:1 (v/v/v), and the polar lipis were developed by chloroform:methanol:acetic acid:water 170:30:20:7 (v/v/v/v). Neutral lipids and polar lipids were stained with iodine, while glycolipids were stained with orcinol. No contaminate lipid bands were observed in the lane of each lipid fraction on the TLC plates (data not shown).

### 3.5. Direct Infusion Tandem MS/MS Analysis

PL and GL fractions were re-dissolved in chloroform/methanol (2:1 v/v). Adequate amount of lipid samples were added into the corresponding solvents according to the detecting ion mode. Chloroform/methanol (2:1 v/v with 1% of formic acid) was for the detection at positive ion mode, and chloroform/methanol (1:2 v/v) was for the detection at negative ion mode. Samples were introduced into ESI-Q-TRAP-MS (QTRAP4000, AB Sciex) with direct infusion at the flow rate of 10 μL/min. Sulfoquinovosyldiacylglycerols (SQDGs) were detected by Q1 scan at negative ion mode, while monogalactosyldiacylglycerols (MGDGs) and digalactosyldiacylglyerols (DGDGs) were detected by neutral loss scan at positive ion mode for the loss of *m*/*z* 162 which corresponding to the loss of a sugar group (galactose). Phosphatidylcholines (PCs) were detected by precursor ion scan for 184.07 (phosphocholine group) at positive ion modes, while other PLs including phosphatidylglycerols (PGs) and Phosphatidylinositos (PIs) were detected by Q1 scan at negative ion mode. Lipids were determined with their calculated *m*/*z*. The fatty acid composition and positional distribution [37,38] were identified with product ion scan of each lipid molecule. Generally, the center position of the glycerol backbone is referred to as *sn*-2 position, and other ends are *sn-*1 and sn-3 positions.

## 4. Conclusions

In conclusion, the polar lipid profile in *Fistulifera* *solaris* JPCC DA0580 was determined with direct infusion ESI-Q-TRAP-MS and MS/MS. Based on our analysis, the chloroplast was demonstrated to play an essential role in the lipid biosynthesis in *F. solaris*. EPA was synthesized on PC in *F. solaris*. In addition, we proposed that the PC-based acyl-editing and head group exchange processes may contribute to the incorporation of EPA into TAGs. To our best knowledge, this is the first report showing the possibility that the PC-based acyl-editing and head group exchange could contribute to the EPA-incorporation into TAG in diatoms. According to this study, the key enzymes in the acyl-editing and head group exchange processes, the PLA, LPCAT and PDCT, can be the future targets for metabolic engineering towards the regulation of EPA content for different purpose.

## Supplementary Files

Supplementary File 1 Supplementary Information (PDF, 24 KB)
