# Supplementary material for: Profiling of Polar Lipids in Marine Oleaginous Diatom Fistulifera solaris JPCC DA0580: Prediction of the Potential Mechanism for Eicosapentaenoic Acid-Incorporation into Triacylglycerol
Source: Mar Drugs. 2014 May 28;12(6):3218–30. doi: 10.3390/md12063218 (PMC4071573; doi:10.3390/md12063218)
Supplement: Supplementary File 1 — Supplementary Information (PDF, 24 KB) [file marinedrugs-12-03218-s001.pdf]

Supplementary Information

Figure S1. The growth curve of *F. solaris* cells cultured in 10 f medium in this study. The cells were harvested at 180 h (black arrow).

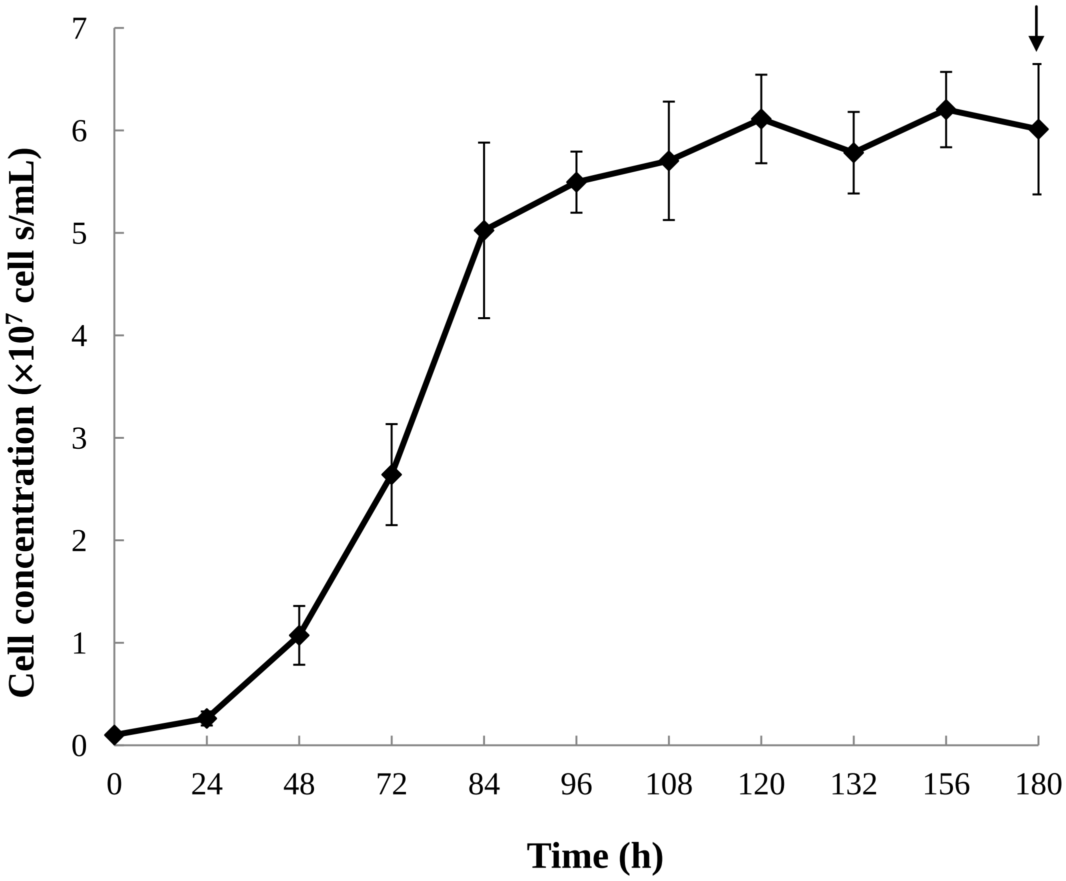

© 2014 by the authors; licensee MDPI, Basel, Switzerland. This article is an open access article distributed under the terms and conditions of the Creative Commons Attribution license (http://creativecommons.org/licenses/by/3.0/).
